# Leveraging routine clinical materials and mobile technology to assess CBT fidelity: the Innovative Methods to Assess Psychotherapy Practices (imAPP) study

**DOI:** 10.1186/s13012-018-0756-3

**Published:** 2018-05-22

**Authors:** Shannon Wiltsey Stirman, Luana Marques, Torrey A. Creed, Cassidy A. Gutner, Robert DeRubeis, Paul G. Barnett, Eric Kuhn, Michael Suvak, Jason Owen, Dawne Vogt, Booil Jo, Sonja Schoenwald, Clara Johnson, Kera Mallard, Matthew Beristianos, Heidi La Bash

**Affiliations:** 1 National Center for PTSD, VA Palo Alto HCS and Stanford University Department of Psychiatry and Behavioral Sciences, 795 Willow Road, Menlo Park, CA 94025 USA; 2 Harvard Medical School and Massachusetts General Hospital, 70 Everett Ave., Chelsea, MA 02150 USA; 3 University of Pennsylvania, Perelman School of Medicine, University of Pennsylvania, 3535 Market Street, Suite 3038, Philadelphia, PA 19104 USA; 4 National Center for PTSD, VA Boston Healthcare System and Boston University School of Medicine, 150 S. Huntington Ave., Boston, MA 02130 USA; 5 School of Arts and Sciences, University of Pennsylvania, 425 S. University Ave., Philadelphia, PA 19104 USA; 6 Palo Alto Veterans Institute for Research, 3801 Miranda Ave., Palo Alto, CA 94304 USA; 7 Suffolk University, 73 Tremont Street, Boston, MA 02108 USA; 8 Stanford University, 401 Quarry Rd, Stanford, CA 94305 USA; 9 Oregon Social Learning Center, Eugene, OR 97401 USA; 10 National Center for PTSD, 795 Willow Road, Menlo Park, CA 94025 USA

**Keywords:** Fidelity, Measurement, Behavioral health

## Abstract

**Background:**

Identifying scalable strategies for assessing fidelity is a key challenge in implementation science. However, for psychosocial interventions, the existing, reliable ways to test treatment fidelity quality are often labor intensive, and less burdensome strategies may not reflect actual clinical practice. Cognitive behavioral therapies (CBTs) provide clinicians with a set of effective core elements to help treat a multitude of disorders, which, evidence suggests, need to be delivered with fidelity to maximize potential client impact. The current “gold standard” for rating CBTs is rating recordings of therapy sessions, which is extremely time-consuming and requires a substantial amount of initial training. Although CBTs can vary based on the target disorder, one common element employed in most CBTs is the use of worksheets to identify specific behaviors and thoughts that affect a client’s ability to recover. The present study will develop and evaluate an innovative new approach to rate CBT fidelity, by developing a universal CBT scoring system based on worksheets completed in therapy sessions.

**Methods:**

To develop a scoring system for CBT worksheets, we will compile common CBT elements from a variety of CBT worksheets for a range of psychiatric disorders and create adherence and competence measures. We will collect archival worksheets from past studies to test the scoring system and assess test-retest reliability. To evaluate whether CBT worksheet scoring accurately reflects clinician fidelity, we will recruit clinicians who are engaged in a CBT for depression, anxiety, and/or posttraumatic stress disorder. Clinicians and clients will transmit routine therapy materials produced in session (e.g., worksheets, clinical notes, session recordings) to the study team after each session. We will compare observer-rated fidelity, clinical notes, and fidelity-rated worksheets to identify the most effective and efficient method to assess clinician fidelity. Clients will also be randomly assigned to either complete the CBT worksheets on paper forms or on a mobile application (app) to learn if worksheet format influences clinician and client experience or differs in terms of reflecting fidelity.

**Discussion:**

Scoring fidelity using CBT worksheets may allow clinics to test fidelity in a short and effective manner, enhancing continuous quality improvement in the workplace. Clinicians and clinics can use such data to improve clinician fidelity in real time, leading to improved patient outcomes.

**Trial registration:**

ClinicalTrials.gov NCT03479398. Retrospectively registered March 20, 2018.

## Background

Decades of research has demonstrated that cognitive behavioral therapies (CBTs) are effective for a wide variety of psychiatric problems and diagnoses [1]. Given this strong evidence base, CBT is cited as a first-line treatment in treatment guidelines for a variety of mood and anxiety disorders [2, 3] as well as for posttraumatic stress disorder (PTSD) [4, 5]. Numerous national, state, and regional mental health systems are implementing CBTs, requiring clinicians to offer them to their clients as clinically appropriate [6, 7]. To date, these efforts have largely focused on provider training and outcome monitoring, with less emphasis on ongoing quality assurance [8, 9].

Decreases in CBT quality can reduce both consumer access to evidence-based psychosocial treatments (EBPTs) and intended symptom improvement [9]. The possibility of “voltage drops” in effectiveness in non-research settings is therefore of great concern to researchers and policymakers [10]. It is important to monitor treatment quality as well as client characteristics and outcomes to learn whether the treatments are being delivered as intended and to understand reasons for potential differences in effectiveness or engagement [11]. Some research suggests that many providers do not implement EBPTs with fidelity [12–14], and a recent study determined that there was no relationship between self-reported primary cognitive behavioral theoretical orientation and observer-rated CBT competence [15]. In light of such research, ongoing quality monitoring and support is a key component in EBPT implementation and sustainability models [16–19]. Furthermore, articulates, policymakers, healthcare administrators, insurance providers, consumers, and other stakeholders need feasible methods of assessing the quality of services that are being delivered.

Efficient, scalable, and non-invasive methods of identifying what occurs in psychotherapy sessions remain elusive, yet they are critical to supporting and evaluating implementation outcomes. Empirically validated fidelity monitoring and support strategies are not yet in place in most healthcare systems [4, 6, 20]. Instruments developed to rate CBT fidelity are typically based on observation. While considered the “gold standard” in psychotherapy outcome research, observation is time- and labor intensive and not feasible in many practice settings [21]. Furthermore, scalable strategies to understand fidelity in practice is necessary, because for many psychotherapies, the link between observer-rated fidelity to session elements is not entirely clear and may differ based on the population characteristics. A recent meta-analysis demonstrated no overall link between observer-rated fidelity and symptom change across a range of treatments and disorders. Notably, though, in the analyses for many studies included in the meta-analysis, aggregated fidelity across the protocol introduced temporal confounds [22]. When analyses were conducted to avoid the confound and establish the temporal precedence of fidelity, two aspects of fidelity, adherence to the protocol, and competence (skill of delivery) in early CBT sessions were associated with subsequent decreases in depression [22]. Later research suggested that fidelity to key aspects of the treatment cognitive processing therapy (CPT; a CBT for PTSD), as opposed to prescribed session elements, was associated with symptom change [21, 23]. Thus, research on session fidelity or quality must establish temporal precedence of these factors to understand relationships between fidelity and symptom change in practice and must also examine strategies that emphasize the quality of delivery of critical treatment elements rather than fidelity to session components.

Researchers have also discovered limitations to existing indirect methods of monitoring fidelity, although they are sometimes used for pragmatic reasons [21]. Clinician self-reports of fidelity (e.g., checklists) appear to correspond only modestly with observer or client fidelity ratings [9, 12, 15, 24–26]. Both our preliminary work and published literature [27] indicate that clinicians (1) perceive adherence checklists as adding to an already high paperwork burden, (2) may not complete them as directed, and (3) may over- or underreport use of prescribed treatment elements. Literature on medical record review, checklists, and clinical progress notes to assess fidelity is mixed [28], and very little is specific to mental health [25, 28, 29]. One study indicated that record review may be a valid method of assessing clinician behavior [30], but research suggests that clinical progress notes convey poor estimates of frequency or intensity of EBPT techniques [30, 31]. Thus, conclusions about the interventions being delivered, and their quality, that can be drawn from self-report or clinical records may be limited.

While manualized CBTs exist for a wide variety of mental health conditions, a common element is the use of worksheets to teach and support client CBT skill development. Diagnosis-specific and transdiagnostic CBT protocols emphasize a set of core CBT elements, targeting clients’ behaviors and thoughts to reduce their symptoms. Both the behavioral and cognitive components of CBTs have been shown to impact symptom change for depression, anxiety, and PTSD [1, 32–35]. Worksheets are used in session to schedule specific behavioral activities and assess their impact on mood, anxiety, or sense of mastery. They are also used to guide one through the processes of cognitive restructuring, which helps clients learn to identify and challenge their maladaptive beliefs and generate balanced alternative thoughts [36, 37]. Assessing the quality of the worksheets that are completed with clinician guidance in sessions may therefore be a pragmatic and valid strategy to assess CBT quality. Thus, using worksheets completed in session to assess treatment fidelity may convey advantages over time-intensive observer ratings and imprecise self-reports of session content.

Another advantage of using worksheets to assess fidelity is that CBT worksheets are accessible and easily transmitted. They are available in paper form and through mobile technology that is accessible to people with disabilities (e.g., hearing- or vision-impaired). The majority of adults in advanced economies, and over half in emerging economies, own smartphones [38, 39]. Mobile applications (apps) are increasingly being used in CBT sessions, making transmission for real-time quality assessment feasible. Worksheets have been adapted for lower levels of literacy, multiple languages, traumatic brain injury, and other factors. Thus, it is possible to tailor worksheets that fit client needs and tailor strategies for collection and rating for different settings. However, the quality and feasibility of data collection may vary across formats and settings. For example, app worksheets may be more legible than handwritten data but may also be briefer or less detailed due to difficulties in typing on a phone. We propose to refine and test a unique method of assessing treatment quality through worksheets generated through routine clinical procedures. Specifically, we aim to (1) develop a scalable general CBT quality measure based on work samples rather than self-report or observation and (2) to evaluate the reliability, validity, and feasibility of different strategies for collecting these materials (e.g., mobile apps vs. paper worksheets).

### Preliminary work

#### Research on worksheet quality

In a preliminary study, we developed a method to assess fidelity to CPT by coding the level of adherence and competence reflected in worksheets that were completed in treatment sessions [40].

#### Reliability

Inter-rater reliability was high (ICC = .68 to .90) for clinician competence on different worksheets. It was adequate to almost perfect (ICC = .55 to .92) on ratings of client skill in completing worksheets independently.

#### Convergent validity

Observer-rated competence on full sessions were not significantly correlated with worksheet ratings (*r*_*pb*_ = .278, *p* = .068), but we found high (Spearman rank) correlations between our worksheet rating method and observer ratings for worksheet-related CBT elements (e.g., cognitive restructuring, *r*_*pb*_ = .823; *p* = .000). Similarly, correlation between observer-rated adherence (*n* = 44 sessions) and adherence scores based on worksheets was low for full sessions (*r*_*pb*_ = .062, *p* = .68) but high for worksheet-related items (*r*_*pb*_ = .808; *p* = .000).

#### Association with symptom change

Overall, our preliminary findings suggest that the worksheet scores were uniquely associated with subsequent symptom change, but client skill was not. Additionally, our preliminary work indicated that symptoms do not predict the quality of worksheets completed in session, although they did predict the quality of worksheets done for homework without clinician guidance [40].

#### Relative advantage

Raters recorded the time to complete ratings (one or more worksheet per session). The mean was 7.04 min (SD = 3.90). Observation and rating of a full session requires 60–75 min or more per session, meaning that this strategy requires approximately 10% of the time required for “gold standard” ratings. The measure had good face validity, and raters viewed worksheets as moderately easy to rate.

## Methods

### CBT worksheet scoring development

Following an iterative process used in previous study, the team’s subject matter experts (who have knowledge of both CBT and the systems in which it is implemented) will develop and refine the scoring system developed in the preliminary study to generalize to common CBT worksheets, with input from end-users (e.g., clinicians, administrators, policymakers, supervisors). We will collect CBT worksheets that are distributed by large mental health systems and CBT training programs and publicly or commercially available books and manuals to identify common elements of CBT worksheets. We will use a rational approach to measure construction and refinement [41, 42], following methods outlined by Vogt and colleagues [35, 43]. We will create a brief, content-saturated measure of CBT quality, with conceptually distinct CBT domains.

We anticipate that minimal changes will be required to make the scoring system developed in the preliminary study applicable to CBT worksheets related to cognitive restructuring, given their similarity to CPT worksheets. Nearly all worksheets that focus on cognitive restructuring contain the following elements: identification of a situation, thought, and feeling/response, questions to challenge beliefs (e.g., evidence for and against), maladaptive thinking patterns, a new/balanced belief, and intensity rating of the emotions and beliefs before and after the cognitive restructuring process. We will develop scoring for common elements of worksheets that track or plan situations, behaviors, emotions, thoughts, and associated intensity ratings.

During the development phase, we will use worksheets collected in previous research. Our team will score these worksheets, and end-users (e.g., clinicians, CBT trainers, quality monitors, administrators, supervisors) will provide feedback on clarity of items, scoring rules, and ease of use, which will help us refine the measure.

Only worksheets that were completed or reviewed and refined in session will be scored to assess CBT quality. Raters will choose items from a list of domains that reflect the instructions and questions on the worksheet to be rated. Items will be scored on a Likert-type scale, and a mean score will be calculated based on all applicable worksheet items. Session scores will be a mean of all worksheets that were completed in session.

We will next examine test-retest reliability, rater agreement on the measure, and the correlation between worksheet ratings and observer ratings of fidelity. We will conduct preliminary analyses to assess associations with symptom change. As we refine the measure and identify content domains, items with higher item-total correlations will take precedence over those with lower item-total correlations, and we will take user feedback into account as well as we refine the measure before entering the validation phase.

### Data collection and research strategy summary

After the development of the scoring system, we will prospectively collect data in a variety of settings, with clinicians (*n* = 120) who have varying levels of experience and expertise with CBT. We will collect data on clinician, client, and organizational characteristics that may impact the assessment of psychotherapy quality. We will collect worksheets, symptom measures, clinical notes with checklists of CBT strategies embedded within them, and therapy session recordings for participating clients. They will be provided to the investigators using secure, Institutional Review Board (IRB)-approved modes of data transmission that have been used successfully in our previous research [44–47].

Participating clients will be randomized into one of two different worksheet formats—app version or paper form (content is the same, mode of completion and data transmission differs). Raters will access the copies of the worksheets, or the mobile app dashboard, and score the worksheets. Clinicians, administrators, supervisors, and clients will be interviewed to assess perceptions of the quality assessment strategies and to determine feasibility, and these data will be analyzed using qualitative strategies.

The variety of worksheets and data capture sources (apps, paper forms) will allow assessment of clinician and client preferences, the degree of detail and completion required for accurate quality assessment. This strategy will allow us to answer several questions through our statistical and qualitative analyses. Analyses will be conducted to answer questions and test hypotheses regarding (1) the reliability and factor structure of the measure, (2) associations between the quality measure and subsequent symptom change (the primary outcome), (3) the concurrent validity of the measure, (4) whether the measure performs better than adherence checklists embedded in clinical notes, (5) whether different formats (apps vs. paper forms) have different concurrent or predictive validity, and (6) the feasibility, burden, satisfaction, and time requirements for the worksheets vs. the adherence checklists.

#### Data collection

We will collect worksheets and clinical note templates (which contain adherence checklists) from clinicians who have varying degrees of CBT expertise in a variety of settings The data sets will include variability in population characteristics and clinician training/education level*.*

#### Clinician inclusion criteria

The clinician inclusion criteria are as follows: (1) be trained (workshop or web-based training and consultation) or in training for CPT for PTSD or CBT that uses worksheets, (2) anticipate at least three eligible clients, (3) be willing to record sessions and provide worksheets and symptom measures to the study, (4) have computer and Internet access, and (5) be willing to use a mobile app on a tablet or mobile device.

#### Client inclusion criteria

Eligible clients (*n* = 360) will be (1) adult outpatients (ages 18 or older), with a clinician diagnosis of primary PTSD (PTSD-Checklist-5 score of 33 or above) [48], a depressive disorder (e.g., major depressive disorder, dysthymia; Patient Health Questionnaire score of 10 or above) [49], or an anxiety disorder (Beck Anxiety Inventory score 22 or above) [50, 51]; (2) those who are willing to allow the team to collect session recordings, measures, notes, and worksheets; (3) those who are able to read and write at a 6th-grade level or above; and (4) those who are willing to engage in CBT.

#### Client exclusion criteria

The client exclusion criteria are as follows: (1) imminent risk for suicide or homicide (requiring hospitalization), (2) in need of detoxification (can be enrolled when substance abuse treatment is not the primary treatment target), (3) active psychosis or manic episode, or (4) cognitive impairments that preclude any participation in therapy.

### Procedure

#### Recruitment and informed consent

Clinicians will be recruited through previously used recruitment strategies including presentation at clinical team meetings (at participating clinics), at CBT trainings, and/or by email. Private practitioners will be targeted through emails to professional organization listservs. After the informed consent, they will complete the clinician measures. They will be provided with an iPod touch, if needed, to use the mobile app. Their clients will be recruited using strategies approved by local systems and IRBs, such as waiting room flyers, whiteboard videos, and/or clinician or intake coordinator referral. Informed consent will be completed by study staff. Clients will then be randomized into one of two worksheet completion strategies: mobile app vs. paper form. Their sessions will be audio-recorded, worksheets will be collected, and they will complete symptom measures (every session for primary problem, every fourth for additional measures) for up to 16 sessions.

#### Worksheet transmission

Clinicians will redact any potential identifiers from worksheets and indicate whether they were completed/reviewed in session or for homework. Paper worksheets will be transmitted by fax or scanned and transmitted electronically (e.g., secure email, shared network drives, and HIPAA-compliant data storage software) depending on local capacity and regulations. The mobile apps will record the scheduled session time and timestamp the worksheets to allow discernment of whether worksheets were done in session. We will use Qualtrics to develop app-based standard cognitive and behavioral worksheets used for depression, anxiety, and PTSD. We will only score worksheets that are done in session to measure quality; scoring of worksheets that are completed for homework will be used to covary client skill in analyses.

#### Collection of adherence checklists

Because templated clinical notes that include checklists of CBT strategies are mandated in some systems and because an alternative hypothesis is that such notes may be an even more feasible, lower-burden strategy for assessing quality, particularly when notes are electronic, we will also collect clinical notes. If extraction from medical records is not possible, copies will be collected in the same way as the paper worksheets and/or using Qualtrics.

#### Collection of session audio

Sessions will be digitally recorded and uploaded to secure servers and HIPAA-compliant data software systems. These strategies have been successful in our previous research, in which over 600 clinicians have provided sessions for full CPT and CBT protocols [45, 52–54]. Clinicians provided similar data when originally trained in the treatments [3, 55, 56].

#### Incentives and retention

Clinicians will receive their worksheet ratings at the end of the study; these will include text with bullet-pointed suggestions for improvement. Clinic-level incentives such a breakfast or lunch tray will be provided once per year, if clinicians keep up with benchmarks for providing data (e.g., 85 + % of required data for currently enrolled clients). If their organization permits, they will receive a gift card for the time they spend transmitting client data (e.g., session recordings, worksheets, symptom measures) for each client. Clients will receive a gift card, if they complete at least four sessions and complete their measures for these sessions.

### Measures and assessment strategy

#### Outcomes and potential mediators

Clients will complete the measure for their primary problem/disorder each session (standard CBT practice). Clients with depression disorder will complete the Patient Health Questionnaire-9 (PHQ-9); clients with anxiety will complete the Beck Anxiety Inventory (BAI) each session, and clients with PTSD will complete the PTSD checklist-5 (PCL-5) every session [48, 57, 58]. We will standardize scores when examining outcomes for analyses but will also look separately at depression, anxiety, and PTSD outcomes in our analyses. Depression, anxiety (PHQ-9 and/or BAI, when not the primary target for treatment), and functioning (Brief Inventory for Psychosocial Functioning; B-IPF) [59] will be measured at the fourth session and posttreatment.

#### Client variables and measures

Secondary analyses will explore whether the rating method is less accurate for subsets of the population. We considered brief tests of literacy, English proficiency, or executive functioning, but because they are orally administered and require assessor training, they are not feasible for clinicians to administer and may cause client discomfort. Instead, we will use variables that capture related and correlated factors including traumatic brain injury and comorbidity (via clinician report) and other demographics via self-report.

#### Client skill

As with our previous work, we will calculate scores for client skill (ability to complete homework sheets; client worksheets will be rated on the same measure). We will report the range of abilities of our sample and use the scores as a covariate in analyses of the association between quality and outcomes.

#### CBT fidelity

We will use the Cognitive Therapy Rating Scale (CTRS) [60, 61], the “gold standard” observer rating scales for CBT for all sessions, and for CPT sessions, we will also use the CPT observer rating scale [21]. Trained postdoctoral fellows and advanced graduate students will rate 10% of the session recordings. They will overlap on 10–15% of these sessions to facilitate reliability analyses. ICCs for observer ratings in our previous studies using these instruments have been high, ranging from .79 to .84 ([45]; Monson et al.: A randomized controlled trial of training and consultation methods to deliver cognitive processing therapy for posttraumatic stress disorder: Impact on patient outcomes, submitted).

#### Clinician and organization variables: covariates

Additional measures will be collected at baseline to explore organization- and clinician-level moderating effects on the association between quality on clinical outcomes (Clinician Demographic Characteristics and Experience Questionnaire, Evidence-Based Practice Attitudes Scale, and Perceived Chrematistics of Interventions and the Implementation climate Assessment) [62–64]. We will assess characteristics that may contribute to quality outcomes [65]: degree, years of experience, gender, age, and prior CBT/ EBPT training.

#### Interviews and assessment of stakeholder perspectives

We will use brief rater surveys and stakeholder interviews (raters, clinicians, 15–20 clients per system, policymakers, and administrators) in each system to assess relevant constructs of the Consolidated Framework for Implementation Research (CFIR) [66] such as perceptions of relative advantage, complexity, design quality/ packaging, readiness for implementation, and inner and outer context factors that might influence implementation. We will assess time required for rating, identify roles that would most likely serve as quality assessors in the different systems (e.g., supervisor, quality assurance staff), assess hourly costs associated with each clinical/quality assessor role, and assess time required for transmitting the worksheets for app vs paper formats. We will assess productivity demands and other demands and responsibilities that may impact use of the assessment strategies. We will follow up to assess perspectives on the contribution of client, clinician, and system characteristics to any variance in quality scores that is identified in our analyses. We will also assess perspectives on use of the mobile apps vs. paper forms, satisfaction, suggestions for refinement, and perceived adequacy of information in the quality measures.

#### Raters and scoring

We will apply the rating system that is refined in the first phase of the study to worksheets that are collected during the entire course of the study. The scoring rules (including decision rules for each session) and scoring algorithms are embedded in an electronic database for app and paper copies. The cadre of raters will include postdoctoral fellows from each system and bachelor’s level research assistants, and some are able to rate sessions and worksheets in Spanish. They will be trained to use the measure and to become familiar with all decision rules, and new decision rules may be identified during this training process. These sessions will be evenly distributed between raters. A subset (10%) will be rated by a second time (1 week after the first rating) to assess test-retest reliability. Furthermore, participating systems will nominate one person (whoever would be in a position to monitor quality) to receive brief training and rate 10 sessions to determine feasibility of training system/clinic-level quality monitors. They will be interviewed regarding potential for implementation within their setting. A randomly selected subset (20%) of the worksheets will be rated by CBT experts and compared with our raters. To score the adherence checklists embedded in the clinical notes, we will score the proportion of CBT elements that are checked off by clinicians.

### Analytic strategy

We will conduct the following analyses:

#### Reliability

We will examine correlations between the subset of worksheets that are rated twice to examine test-retest reliability. Next, we will compute estimates of internal consistency reliability for hypothesized CBT domains (e.g., identifying thoughts and feelings, cognitive restructuring, behavior planning and tracking) in the form of Cronbach’s alphas and retain domains with an alpha of .70 or above. We will calculate intraclass correlations (ICCs) for the total measure and for each content domain, using a random effects model to estimate the reliability of rater judgments [67]. Per established conventions and prior research [68], we will apply a minimum criterion ICC = .60 (“good”) [69] to indicate acceptable agreement. We will also compare bachelor’s level raters’ scores to expert rater scores to assess the degree of agreement, to determine a level of expertise required for rating.

#### Concurrent validity

We will compare our strategy to the “gold standard” fidelity measure and clinical progress notes alone. We predict that ratings on our quality monitoring method will be highly correlated with existing [3, 17] objective and reliable observer ratings and that they will be more accurate than the ratings based on the checklists embedded in the clinical notes alone [2, 21, 70, 71]. We will examine Spearman rank correlations [72] between our method and independent observer ratings for the overall measure, for each subscale and for conceptually corresponding items. Further, we will conduct cross-validation borrowing the idea of checking internal validity of prediction models in statistical learning [73].

#### Factor analysis

We will conduct an exploratory factor analysis to examine the factor structure of items within each worksheet. It is possible that quality may differ across different elements/domains of CBT, but it is also possible that CBT quality is better represented as a composite variable (some areas are of higher quality than others and that summing across them still provides good information about overall quality). Thus, we will explore the possibility of multiple factors or domains (e.g., cognitive restructuring), and will also evaluate the extent to which the components are best represented as a latent variable, that is, a general tendency to provide high-quality CBT that is reflected in high scores across different CBT components.

#### Clinical outcome predictions

We expect that clients whose sessions receive higher quality ratings will exhibit larger decreases in symptoms compared to clients whose sessions had lower ratings. Based on previous research suggesting that it is important to assess fidelity in early sessions to avoid temporal confounds between process variables and outcomes [22, 68, 74, 75], we expect that quality in earlier sessions will more accurately reflect the contributions of session quality and clinician skill (rather than client factors) than that in later sessions. Primary analyses will focus on quality in sessions 1–6. We will cross-validate prediction models as a way of checking internal validity (generalization error).

#### Analyses

We will first examine distribution of key variables for skewness, variability, and outliers and apply appropriate transformation or other strategies to address non-normality (e.g., robust estimation procedures) as necessary. Symptom scores from instruments that reflect the target problem for each client (e.g., depression, anxiety, or PTSD) will be standardized, and the standard scores will be used as the dependent variable (DV). We will also conduct secondary analyses with the measures for each target problem and with the functioning measure as DVs. The study will produce hierarchical data with repeated measures (Level-1) nested within clients (Level-2) nested within clinicians (Level-3). To evaluate change in client outcomes, we will conducted multilevel regression (i.e., mixed-effects regression, hierarchical linear modeling) growth curve analysis, which offers numerous strengths for analyzing change in nested data, including efficiency in handling missing data, powerful and accurate estimation procedures adjusting for clustering, and modeling flexibility (e.g., allows for the inclusion of continuous or categorical, time-invariant or time-varying covariates and predictors). We will employ strategies to check the sensitivity of analysis results due to missing data (e.g., pattern mixture modeling) [76]. We will analyze the intent-to-treat sample [77] and completer samples. First, multiple unconditional change models (i.e., change without predictors) will be evaluated to determine the most reliable and powerful way to analyze change (e.g., linear or non-linear change; modeling time as time since baseline or as session number) and determine the most appropriate variance-covariance structure, considering, among others, the autoregressive structure.

The first set of multilevel regression analyses will evaluate trajectories of outcomes over the entire course of therapy or over the course of several sessions. We will examine the influence of potentially significant covariates (e.g., baseline scores, setting, veteran status, client skill, and other clinician, setting, or client characteristics) and include in exploratory analyses. Given the small number of systems, the system will be entered as a potential covariate to assess and adjust for its influence, but we will conduct an exploratory analysis with the system entered at Level-4.

The next multi-level regression analyses will examine the session-by-session associations between quality and symptoms. Lagged multivariate models will allow for the inclusion of two outcomes (quality and symptom change) in the same model [78–80]. Evaluation of the cross-lagged paths will provide information about temporal precedence in the relationship between the two variables across time. We will also account for overall increases and decreases in the model by including time as a predictor of each outcome.

We will next determine whether the different quality measures or worksheet formats differ in terms of how strongly they are associated with subsequent symptom change. Since clients will be randomized into worksheet format (app vs paper form), a dichotomous variable with paper form as the reference will be entered at Level-2 in the analyses described above to examine whether format predicts symptom change. We will explore potential interactions with target problem (PTSD, depression, anxiety). We will examine and compare the effect sizes and the proportion of variance accounted for by the worksheet measure and the adherence checklist. We will also compare them for app vs. paper form worksheets. A medium or large effect would be clinically meaningful in the context of the pragmatic goals of this project, particularly because the reliable change index for each of our outcome measures corresponds to a large effect size [48, 49, 58]. On the other hand, if associations with outcomes differ only slightly, we would recommend selection of a quality assessment strategy based on specific programs’ needs or goals.

#### Comparisons of quality measures (worksheet vs. adherence checklists) and worksheet formats

If self-reported adherence (as assessed through the checklists embedded in clinical notes) is highly correlated with worksheets and/or the gold standard observer ratings, and if they are strongly associated with outcomes, the checklists alone may be deemed sufficient for quality assessment, because the notes can already be accessed by quality monitors in each system. First, we will use the Fisher *r*-to-*z* transformation to calculate a value of *z* that can be applied to assess the significance of the difference between two correlation coefficients. This analysis is conducted to assess whether a significant difference exists between correlations with the gold standard observer ratings. If there is no significant difference, this would indicate that the worksheet quality scoring strategy does not yield an advantage or disadvantage in terms of its correspondence with gold standard observer ratings. We will also use this strategy to determine whether a difference exists between correlations with worksheet formats (app vs. traditional paper form) and observer ratings [48].

#### Sample size justification and power calculations

In light of the project’s emphasis on the development of pragmatic measurement strategies that predict changes in the target problems, we powered the study to test for predictive validity. Calculations of a design effect (measure of how the design effects the standard error of the parameters) [81, 82] accounted for clustering. We computed ICC with repeated observations of clients at the clinic (*r*_*pb*_ = .01), clinician (*r*_*pb*_ = .01), and client (*r*_*pb*_ = .10) level in our prior research, assuming three clients per clinician, and six clinicians per cluster, and six observations per client, yielding a design effect of up to 2.04. We based the estimated number of observations per client to conservatively account for potential 37% client-level attrition and missing data during the treatment. For the growth curve analyses to assess whether the quality measure predicts symptom change (our primary analysis), the probability of a type 1 error (alpha) at two-tailed .05 power exceeds .80 for an effect size of *r* = .30, which corresponds with small-to-medium effects of fidelity on symptoms in prior CBT fidelity research [68, 83]. We also projected a sample size to be sufficient to conduct separate analyses and test for interactions with disorder (PTSD vs. depression vs. anxiety), app vs. paper form, and other variables of interest, and detect an effect size of .50, a medium effect.

## Discussion

To date, there is no feasible and easily adoptable way to effectively assess clinician fidelity of CBTs. This study aims to refine and test a unique method of leveraging data generated through routine clinical procedures to assess quality to (1) develop a scalable general CBT quality measure based on work samples rather than self-report or observation and (2) to evaluate the reliability, validity, and feasibility of different strategies for collecting these materials. Our method poses little additional burden to clients or clinicians. Because it uses routine clinical materials, it allows for a less “invasive” review of a random selection of sessions by a quality monitor or supervisor. Since treatments are sometimes modified in practice settings [8], it will be designed to apply to modifications to worksheets that are adapted to meet client needs. To our knowledge, this will be the first instrument of this nature to be developed and validated. It will thus fill a critical gap in the field and has the potential to make large-scale quality monitoring in research and practice settings more feasible and efficient. Scalable methods of quality monitoring are vital to efforts to study and promote the implementation of EBPTs [84, 85]. This study has strong potential to impact fidelity monitoring strategies for a variety of CBTs due to shared elements across CBTs [36]. A less burdensome method can dramatically increase feasibility of ongoing quality monitoring. Validation across platforms (mobile app and traditional paper forms) and data on the feasibility and acceptability of each of these platforms is an additional innovation that will yield actionable data to inform implementation. This in turn can promote continued consumer access to high-quality delivery of CBT and other EBPTs [9, 16, 22], and protect the significant investment in implementation across multiple systems.

## Abbreviations

app: Application
BAI: Beck’s anxiety inventory
CBT: Cognitive behavioral therapy
CPT: Cognitive processing therapy
CTRS: Cognitive therapy rating scale
EBPT: Evidence-based psychosocial treatment
HIPAA: Health Insurance Portability and Accountability Act
ICC: Intraclass correlations
IRB: Institutional review board
PCL-5: Posttraumatic stress disorder checklist-5
PHQ-9: Patient health questionnaire-9
PTSD: Posttraumatic stress disorder
SD: Standard deviation
VA: Veterans affairs
